# AI in Mental Health: Transforming Diagnosis and Management of Depression and Anxiety

**DOI:** 10.1002/hsr2.72316

**Published:** 2026-04-14

**Authors:** Tilyan Kambar, Sara Tariq, Saman Shahzad, Hajra Sana Sultan, Araj Naveed Siddiqui, Fakiha Ahmed Shah, Hamna Khanani, Ayesha Khan, Hussain Haider Shah, Sameer Abdul Rauf, Muhammad Abdul Wasay Zuberi, Radeyah Waseem, Muhammad Sheheryar Hussain, Md Ariful Haque

**Affiliations:** ^1^ Department of Medicine Ziauddin University Karachi Pakistan; ^2^ Department of Medicine Ziauddin Medical College Karachi Pakistan; ^3^ Department of Medicine Jinnah Sindh Medical University Pakistan; ^4^ Internal Medicine Dow University of Health Sciences Karachi Pakistan; ^5^ Liaquat National Medical College Karachi Pakistan; ^6^ Department of Public Health Atish Dipankar University of Science and Technology Dhaka Bangladesh; ^7^ Voice of Doctors Research School Dhaka Bangladesh

**Keywords:** anxiety, artificial intelligence, chatbots, deep learning, depression, diagnostic tools, machine learning, mental health, psychiatry, wearable devices

## Abstract

**Introduction:**

Mental health disorders, especially depression and anxiety, are major contributors to the global disease burden. Traditional psychiatric methods can be time‐consuming and often struggle with accurate diagnosis and effective treatment. Artificial intelligence (AI) has the potential to improve diagnostic precision and streamline the management of mental health issues.

**Methodology:**

This review investigates AI's role in addressing mental health challenges, with a focus on anxiety and depression. Relevant literature was sourced from Medline, Google Scholar, and PubMed, using keywords like “artificial intelligence,” “mental health,” “depression,” and “anxiety,” emphasizing studies involving Asian populations. The search included English‐language articles, which were screened based on titles and abstracts.

**Discussion:**

AI applications in psychiatry, including chatbots and wearable devices, enable early detection and individualized care. Machine learning and deep learning models that use data from sources such as social media and sensors assist in diagnosing and monitoring mental health conditions. Although these tools provide valuable support, ethical concerns related to privacy, algorithmic bias, and limitations in detecting suicidal ideation need to be addressed.

**Conclusion:**

AI shows promise in transforming mental health care by increasing diagnostic speed, accuracy, and accessibility. Despite existing challenges, particularly around ethical considerations and acceptance in older populations, further research and careful regulation could allow AI to complement human‐centered psychiatric care. Future studies should work to maximize the benefits of AI while preserving the critical human connection in mental health services.

## 1 Introduction

Psychiatric diseases are acknowledged as a primary contributor to the worldwide burden of disease, accounting for 4.9% of global disability‐adjusted life‐years (DALYs) and an age‐standardized DALY rate of 1566.2 per 100,000 individuals [1]. In the last 10 years, there has been a notable acceleration in psychiatric challenges, particularly depression and anxiety. The COVID‐19 pandemic has played an important role in this significant increase [2]. Psychiatric disorders have caused an impact on individuals and society, leading to physical morbidity and premature mortality [3]. One prevalent mental illness is depression, often known as major depressive disorder. It describes a low mood or aversion to tasks of everyday living that lasts for at least 2 weeks [4]. The world's 3.8% population is affected by depression, equating to approximately 280 million individuals globally [4]. On the other hand, anxiety disorders are becoming more prevalent with an estimated incidence of 4% globally [5, 6]. That equals around 301 million people [5]. Common symptoms include an overwhelming sense of terror and anxiety or avoidance of recognized dangers that may interfere with everyday life [6].

Many of the mental health challenges can be overcome by the implementation of artificial intelligence (AI) in the psychiatric field. AI has largely revolutionized the world. Applications for AI in all fields of medicine are steadily advancing. The integration of AI in healthcare allows the manipulation of extensive data and knowledge to assist doctors in their everyday tasks and in resolving complex clinical challenges [7]. Nevertheless, the field of psychiatry has been cautious in embracing AI‐based technologies [8].

Traditional psychiatry is strictly focused on patient‐psychiatric interaction [9]. Psychiatrists make decisions on the subjective basis of the patient's course of symptoms, cognition, and emotional status [10]. Doctors assess the mental well‐being of their patients through keen observations in real‐time sessions and by seeking further information from patients' friends and family [9]. However, the clinical assessment process is often hindered by the minimal amount of time allocated for healthcare [10]. AI tends to provide complementary support to clinicians by streamlining functions that do not necessarily require a “human touch”. This helps doctors to concentrate more on providing empathetic care to their patients, ultimately “humanizing” mental health care [11]. AI has a probable prospective in improving diagnostic accuracy by assisting in clinical reasoning and judgment processes, thereby further augmenting clinical intuition [12]. Additionally, AI aims to leverage the mechanistic knowledge of mental disorders to enhance prediction, detection, treatment, and interventions [13, 14].

Despite posing a great burden on global health, the accurate diagnosis of psychiatric illnesses, which is essential for effective treatment and support, remains a formidable challenge. However, newer innovative methods like AI can help to diagnose psychiatric illnesses, such as depression and anxiety. This article summarizes the impact of AI in the scope of psychiatry, its role in the diagnosis and management of depression and anxiety, ethical considerations, and the advantages and drawbacks of its implementation in psychotherapy. It further discusses the ongoing limitations of implementing AI and e‐mental health and discusses its future in mental healthcare.

## 2 Methodology

This narrative review addresses how chatbots and artificial intelligence are affecting the increasing issue of mental illness. Mental health issues seriously impact our society, necessitating urgent attention. A thorough examination of the literature was carried out on Medline, Google Scholar, and PUBMED databases from inception till February 2025. The following keywords were used: “artificial intelligence,” “mental health,” “depression,” and “anxiety.” Articles written in languages other than English were omitted. Included were data from contemporary and historical publications as well as reviews of mental health issues, particularly affecting the Asian population. The inclusion criteria were followed in the screening and review of the paper titles and abstracts.

## 3 Discussion

Before exploring the applications of artificial intelligence in psychiatric disorders, it is important to understand the existing burden of mental health disorders, such as depression and anxiety, across different populations. Establishing the epidemiological context provides a clearer picture of the scale and variation of these conditions globally and regionally, particularly in Asia, where sociocultural and healthcare disparities influence both diagnosis and management. This understanding lays the foundation for appreciating how AI technologies can be tailored to address these mental health challenges effectively.

### 3.1 Epidemiology

Only a few studies on depression have been carried out in older Asian individuals, but the findings differ significantly. Using standardized questionnaires and individual interviews, [15] used the 10‐item Centre for the Epidemiological Studies Depression Scale (CES‐D) in Hong Kong to diagnose depressed symptoms in Chinese psychiatric patients. The patients ranged in age from 70 to 90 years. Thirty older persons from a community center, 54 older adults from the general public, and 30 older adults with depressive symptoms made up the sample. A study by [16] from 1995 included a sample of 298 Chinese patients over 60 years of age and a validation sample of 100 older Chinese patients. The study was based on the Chinese version of the Hospital Anxiety Depression (HAD). Two psychiatrists scored the validation sample using the Clinical Interview Schedule on a 0–4 scale. For mental illness, a score of more than two was deemed noteworthy. According to the results, 78 (26%) patients had an anxiety score of 3 or greater. Amongst them, the female patients had higher anxiety scores.

Studies across Asia highlight risk factors that contribute to the development of depression in older adults, including lower educational attainment, female gender, marital difficulties, economic strain, advancing age, family conflict, loneliness, and deteriorating physical health. Chronic medical conditions further increase vulnerability to depression.

Research in Nepal suggests that depression among older adults is frequently undetected and untreated, largely because competing health priorities overshadow mental‐health care. Studies conducted in urban areas and senior living facilities found that depressive symptoms were influenced by chronic illnesses and complex care needs. These studies used the 15‐item Geriatric Depression Scale (GDS‐15), and findings indicate that depression is a substantial yet under‐recognized issue among Nepalese older adults [17].

In China, studies have identified advanced age, loneliness, functional impairment, and female gender as key factors associated with depressive disorders in later life. Anxiety symptoms have also been documented in institutionalized older adults in Thailand, and concerns about suicide risk highlight the need for improved monitoring and mental‐health support services [18, 19].

Self‐reported depression in South Asian countries has shown that many older adults experience symptoms at some point in life; however, formal diagnosis remains low due to stigma, underreporting, and limited access to care. Similar patterns have been noted in Iraq, where older adults often experience depressive episodes without receiving adequate treatment [20].

**Figure 1:**
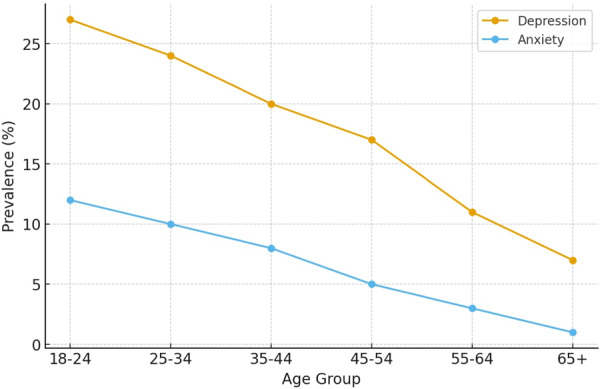
Depression and anxiety trends by age group.

The age‐group trend indicates that both depression and anxiety decrease steadily from young adulthood to old age. Depression rates decline from about 27% among individuals aged 18–24 to roughly 7% in adults aged 65+ , while anxiety decreases from about 12% to nearly 1%. (Figure 1) [5]. These global age‐trend findings differ from many Asian studies showing higher depression in older adults. Therefore, the high late‐life depression recorded in many Asian studies likely reflects population‐specific factors such as chronic illness, reduced social support, socioeconomic challenges, and limited mental‐health service access.

**Figure 2:**
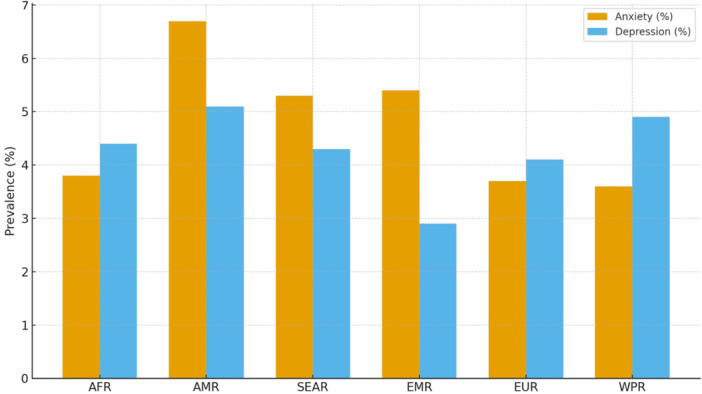
Depression and anxiety regional prevalence by WHO.

According to the WHO regional comparison shown in the Figure 2, the global prevalence of anxiety and depression varies moderately across regions, with anxiety ranging from approximately 3.6% to 6.7% and depression from about 2.9% to 5.1%.

### 3.2 Mental Health Disorders: Key Insights and Implications

According to the World Health Organization (WHO), mental health makes up an important component of one's health; it allows a person to be able to cope with stress, discover their capacity, learn and work efficiently, and contribute to the community [21]. According to a survey, depression and anxiety are ranked first and sixth in the world, respectively, adding to the global disability burden [22]. A study characterized anxiety disorders into panic disorder, generalized anxiety disorder (GAD), social anxiety disorder (SAD), obsessive‐compulsive disorder, and post‐traumatic stress disorder [23].

Depression and anxiety are among the most common ailments that plague a community. They are primary conditions that usually present with certain somatic symptoms, which include general fatigue, agitation, and cognitive disabilities such as impaired memory, indecisiveness, and poor thinking. Other symptoms include impaired personality and atypical anger. Muscle aches and headaches are common symptoms along with gastrointestinal, cardiovascular, and respiratory manifestations. The patient also combats genitourinary symptoms, such as loss of libido and difficulty in micturition. Neurological manifestations might include dizziness, blurred vision, and vertigo [24].

Augmentation of mental illnesses, such as depression and anxiety, usually occurs due to the stigma associated with them. This further leads to harmful outcomes for the individual and can discourage the patient experiencing mental health challenges from seeking help [25]. A study reported that anxiety mostly co‐occurs with substance abuse. Three etiological models have been proposed that show an association between the two and assist in their management. The first pathway states that anxiety causes substance abuse, while the second pathway states that anxiety results from the usage of these substances, while the last one appreciates common factors that lead to both. The first model elaborated that anxiety symptoms are typically relieved by alcohol, cannabis, or opiates, and patients use them as coping strategies, while the second model states that many of these substances result in anxiety symptoms or anxiety resulting from their withdrawal. Childhood trauma and personality deficits apprehend the last model [26].

The mental health of women in their perinatal period is greatly affected, as reported by some studies. These women experience postnatal depression, high‐risk pre‐term birth, and fear of childbirth. Suicide is also an excruciating symptom [27]. Antepartum depression usually results in adverse health implications, such as poor nutrition, increased substance abuse, and postpartum depression. It typically remains unrecognized as priority is given to preventing deaths and obstetric complications rather than mental stressors [28]. Socioeconomic status, chronic poverty, gender inequality, and a poor level of education in women are major factors that increase the risk of mental disorders in women living in underdeveloped or developing countries [27].

According to DSM‐IV and ICD‐10, to classify a condition as major depression, a state of displeasure, loss of interest, and depressed mood should be present. Late‐life major depression results in changes in the body, which include hypercortisolemia, obesity, decreased bone density, and increased risk of developing type 2 diabetes and hypertension. According to a study, geriatric depressive disorder entails the following classification: (i) major depressive disorder, (ii) minor depressive disorder, (iii) dysthymic disorder, (iv) bipolar I disorder, and (v) adjustment disorder with depressed mood.

Patients with general medical conditions also develop depression. These include viral infections, endocrinopathy, malignant disease (leukemia, lymphoma, and pancreatic cancer), cerebrovascular diseases (lacunar infarcts, stroke, and vascular dementia), myocardial infarction, and certain metabolic disorders [29].

According to a study, depression adversely affects the outcome and prognosis of hypertension. Geriatric depression was also discovered to occur as a result of cardiovascular disorders (CVDs) and other chronic conditions, which often attenuate one's self‐rated health and cognitive function. It was further uncovered that the risk of coronary heart disease and myocardial infarction is increased by 1.5–2.0 times and 1.5–4.5 times, respectively, due to depression [30].

Co‐occurrence of different mental health issues leads to decreased well‐being and an increased burden on healthcare [24]. People with mental ailments are more prone to develop obesity and metabolic disturbances, which greatly contribute to developing CVDs and type 2 diabetes. Moreover, people with mental health disorders are also more likely to smoke and have an improper diet with minimal physical activity; these factors increasingly contribute to the development of critical conditions [31].

### 3.3 Artificial Intelligence‐Driven Approaches to Diagnosis and Management of Mental Health Disorders

In the case of mental diseases, prompt diagnosis and appropriate treatment are crucial. When discussing the treatment, various approaches are accessible. According to one article, pharmaceutical therapy, which includes antidepressant and antianxiety medications, is essential to the treatment of depression and anxiety. Furthermore, psychological therapies such as internet‐based interventions, mindfulness‐based therapies, progressive muscle relaxation training, and cognitive behavioral therapy (CBT) are crucial in reducing psychological issues. Physiotherapy is an additional or alternative kind of treatment to medication and psychological counseling. Previous research has validated the effectiveness of neuromodulation in treating depression, and people with mental health issues can benefit from it due to its favorable effects and low side effects [32].

According to another research, exercise appears to be the most beneficial treatment option for posttraumatic stress disorder, unipolar depression, and treatment‐resistant depression. Yoga has been demonstrated to be useful as a standalone or adjunct therapy, notably for depression. It helps treat anxiety disorders, especially panic disorder, as an additional therapy. Although the findings are mixed, tai chi and qi gong may be useful as additional treatments for depression. Mindfulness‐based meditation is useful for depression, both as an independent remedy and as an adjuvant therapy, with a half‐life of 6 months or longer. Positive outcomes are less common in persons with anxiety disorders, but evidence still encourages the use of additional treatment. Mindfulness‐based interventions appear to have no adverse consequences, and their health‐related benefits justify using them as an added therapy for people suffering from anxiety and despair [33]. A randomized control trial discovered that, in addition to the previously listed treatment options, nutrition can be an important component in the management of mental health disorders. A nutritious diet that includes whole grains, fruits and vegetables, and fish was connected with a lower risk of depression in adult participants [34].

According to a study, Facebook Messenger and mobile apps can be used to communicate with Woebot, an autonomous chatbot. It offers tools for automating cognitive behavioral treatment (CBT). This technique was developed to monitor symptoms and manage depressed and anxious episodes by teaching skills such as identifying and correcting cognitive errors. Similarly, in a randomized controlled trial, 70 patients were assigned to either Woebot or e‐book reading for depression. The Woebot group reported much lower levels of depression than the e‐book group did [35].

Mobile applications, such as those that use smartphone data, have been utilized to track and monitor symptoms of depression and anxiety, with machine learning models often incorporating data such as audio, GPS, or light sensors [36].

According to a randomized control trial, Tess is an additional resource for helping people overcome emotional discomfort through text messaging. It can be used as a contact number. This software offers emotional wellness coping skills and enables therapeutically comparable interactions between a user and a psychotherapist [37].

It was noted in a different study that patient health data tracking and evaluation constitute the primary uses of wearable technology. However, there have been notable advances in the detection and treatment of nervousness and depressive disorders due to wearable technologies and artificial intelligence. In addition to enabling more individualized care for those who suffer from such diseases, it may result in a more accurate and prompt diagnosis of anxiety and depression and the development of prophylactic measures for populations at risk of developing these disorders [38].

A person's mental health must be understood and assessed using a variety of techniques. Studies have demonstrated differences between the general population and those suffering from depression. These can include non‐verbal behaviors like voice characteristics, facial expressions, and language use (verbal or textual), biochemical markers like blood oxygen consumption in the brain, and peripheral physiological signals like skin conductance, heart rate, and neurotransmitters. The traits that separate the two groups, those with and without depressive disorders, are the foundation of basic AI classifiers. Thus, AI can develop effective predictive models for automated depression diagnosis by utilizing these distinguishing features in machine learning.

Gavrilescu et al. [39] suggested using the Facial Action Coding System to analyze facial expressions to determine depression levels. The experiment's accuracy rate in identifying depression was 87.2%. Moreover, it has been suggested that the length, intensity, lip movement, and absence of spontaneous smiles can provide useful patterns for the identification of depressive disorders. The identification of depression by facial expressions has advanced significantly in the last few years. Pupil variations are now considered effective face features. Faster pupillary responses, for instance, were seen to reflect a good, healthy control in a recent study. Under some circumstances, delayed pupil dilation responses are seen in depressed subjects [35].

### 3.4 Applications and Ethical Considerations of Artificial Intelligence in Mental Health

OpenAI, an artificial intelligence software designed for a variety of uses, created the Chatbot Generative Pre‐trained Transformer (ChatGPT). It operates by using written or spoken inputs that are configured to provide relevant generated responses by AI [40]. It is trained to translate large amounts of text data into text that looks like writing by humans and is built on transformer architecture. AI and chatbots have advanced significantly over the years, and they are valuable in a variety of industries, including healthcare, education, and customer service [41]. AI utilizes various techniques such as machine learning (ML), deep learning (DL), and natural language processing (NLP). Large language models (LLMs) are a type of deep learning technique that generate text‐based content based on massively large data sets. NLP is a subfield of AI that focuses on the interaction between computers and humans through natural language by understanding, interpreting, and generating human language [42, 43, 44].

Artificial intelligence is a powerful tool that has transformed many aspects of human life with its versatile applications. The role of AI in medicine has truly made a great advancement in the field. It can be used as a means of diagnosing and correctly identifying potential health risks. AI has revolutionized the medical industry by processing large amounts of data, accurately making early diagnoses of diseases, and keenly identifying patterns in scans and tests that could have been easily missed by the human eye, eventually leading to more tailored treatments for patients according to their needs and preferences [45]. AI also aids in monitoring the patient's health by keeping track of their record. Hence, it can provide a more personalized insight into their condition with early detection of chronic conditions.

Mental health practitioners have a more hands‐on approach with their patients in treating their patients by forming a relationship with patients and personally observing their emotions and behavior. An individual's unique bio‐psycho‐social profile better represents their overall mental health [46]. With the identification of biomarker levels, creation of screening tools, and creation of risk models to ascertain a person's propensity for or likelihood of developing any mental illness, AI is still subjectively involved in the understanding and prediagnosis of a variety of mental illnesses [47].

AI has improved the way that people with mental illnesses are currently treated. Specifically, AI algorithms have made it possible for practitioners to understand the population‐level prevalence of mental illnesses by identifying risk and protective mechanisms. They have also made it possible to monitor treatment progress and even patient adherence to prescribed regimens. Finally, AI has made it possible to deliver remote therapy sessions and provide self‐assessments to determine the severity of the condition in the form of self‐evaluation. Most importantly, AI has made it possible for practitioners to concentrate on the human aspects of medicine, something that was previously only possible through clinician‐patient relationships [48].

The Oxford Martin Institute and Deloitte partnership estimated that, in the next 10–20 years, artificial intelligence could evolve to the point when 35% of United Kingdom occupations will be completely automated [49]. According to another research, several external factors, including the price of automation technologies, labor market demand, cost, advantages of automation beyond labor replacement, and social acceptance, could keep the percentage of jobs lost to just 5% or less [49, 50].

Although AI has substantial advantages, it still has reservations with the geriatric population. The use and acceptance of AI have been limited because the older population has limited interaction with social interfaces altogether, and acceptance of the rise of artificial intelligence over human intelligence has restrained its use on the older population, who otherwise have a higher prevalence of conditions like anxiety and depression.

The integration of AI into mental health care presents several ethical challenges, as identified in recent medical literature:

1. Informed consent and transparency: AI systems often function as “black boxes”, making it difficult for patients to comprehend decision‐making processes. This hence veils the traditional notions of informed consent and transparency in healthcare [51].
2. Privacy and data security: The use of AI in mental health care makes the collection and analysis of extensive personal data essential, which raises significant concerns about patient privacy and data security. It is crucial to ensure that AI systems uphold robust data protection standards [52].
3. Bias and fairness: AI systems, including those used in mental health, rely heavily on large data sets for training. If these data sets reflect societal biases (such as gender, race, or socioeconomic status), AI systems may perpetuate or even exacerbate existing disparities in mental health care. These biases in AI models can lead to incorrect or unfair predictions, resulting in unequal treatment for marginalized groups.
4. Human–AI interaction and empathy: While AI can simulate therapeutic conversations, it lacks genuine empathy and understanding. Over‐reliance on AI for emotional support may lead to diminished human interaction, which is vital for effective mental health care [53].
5. Accountability and responsibility: Determining who is responsible for AI‐driven decisions—whether developers, healthcare providers, or institutions—is a complex ethical issue, especially when AI recommendations lead to adverse outcomes [54].

Recent incidents have underscored these concerns. For example, an AI chatbot's harmful advice led to a user's suicide, highlighting the potential risks of AI in mental health [55]. Additionally, the Vatican has issued guidelines cautioning against substituting human connections with AI, emphasizing that AI should complement human intelligence rather than replace it [56].

Scholars and ethicists continue to debate the balance between preferring AI's benefits in mental health care and overlooking its ethical risks. Ongoing research and discourse are essential to navigate these challenges responsibly.

AI has significantly transformed healthcare, yet ethical challenges remain, as patient–clinician relationships, built on empathy and human connection, remain central. For example, deep learning algorithms used in image analysis can accurately diagnose cancer, but they are unable to explain or interpret the reason behind the diagnosis. Furthermore, algorithm bias can affect machine learning systems used in the healthcare industry, potentially leading to the prediction of a condition's increased likelihood based on factors such as gender or ethnicity, which would not otherwise be the cause. It is safe to conclude, then, that concerns about authorization, privacy, accountability, and openness may arise when AI is used in healthcare [57].

### 3.5 Exploring the Diverse Advancements of Artificial Intelligence in Mental Health: Chatbots, Machine Learning, and Deep Learning Models

Mental health issues are increasingly prevalent in modern societies, presenting significant challenges to healthcare systems worldwide. Leveraging artificial intelligence offers promising avenues for addressing these challenges. With the proliferation of social media and mobile technology, coupled with advancements in AI, there exists a unique opportunity to tackle mental health problems effectively [58].

Machine learning plays a vital role in AI, particularly in disease classification. Research has examined the potential of machine learning models, such as support vector machines, in diagnosing conditions like depression and anxiety [59]. For example, an Android app was created in conjunction with a centralized server system to collect objective smartphone data, such as ambient audio, GPS location, screen condition, and light sensor information. Adult Canadians taking part in a 2‐week observational research study provided the data for this investigation. Predictive models for depression, generalized anxiety disorder, and social anxiety disorder were constructed using high‐level variables that were taken from this data [60].

Researchers have employed a variety of data sources to identify anxiety and depression; the most popular sources are publicly accessible databases and data from hospitals or research facilities [61]. However, because the procedure was so laborious, creating new data sets appeared to be less common. The most common data types that were studied were text data from social media and EEG signals, then audio and face video features [61]. This underscores a preference for easily accessible data sources in mental health detection efforts, with a notable focus on depression compared to anxiety disorders.

Methodological approaches vary, with recurrent neural networks (RNNs) employed for audio feature analysis recognized to have limitations in generalizing across different data sets [62]. While enriching deep models with additional features has shown promise in improving detection accuracy, it also increases computational complexity. Additionally, integrating visual features into models has resulted in significant performance improvements, although concerns about potential invasiveness for patients have been raised [62].

Another study highlighted the effectiveness of integrating geometric and facial marker features in enhancing model performance [63]. Yet, caution was advised against overfitting when incorporating polynomial feature sets, as it led to decreased model performance [64]. Moreover, a different study emphasized the superiority of their hybrid model, which fused text and semantic features with audio and visual data for depression detection, leading to enhanced performance compared to existing methods [64]. These findings collectively underscore the potential of AI and machine learning in revolutionizing mental health diagnosis and treatment.

A few writers have carried out reviews of studies on the identification of depression, offering valuable perspectives on diverse techniques and strategies. One evaluation, for example, highlighted a move towards using deep models for speech depression recognition by focusing on the use of deep learning techniques with speech signals [65]. To improve our understanding of the fundamental mechanisms underlying speech in depression, they suggested integrating clinical data and supporting the integration of several modalities for more comprehensive depression analysis in subsequent studies [66]. In contrast, a different research study recommended particular methods for visual feature extraction and focused on classic machine learning techniques with visual face signals for depression identification. But in contrast to our review, which covers a wider range of data sources and feature fusion strategies, their attention is limited to conventional machine learning techniques and visual facial signals [67, 68].

In another vein, a review study explored detection of depression based on text analysis from social media, finding that classifiers and probabilistic classifiers were commonly used for this purpose [69]. These findings differ from ours but underscore the diversity of approaches in the field of depression detection. Another review study focused on depression sign detection using social media, highlighting specific platforms and prevalent linguistic feature extraction methods and classifiers [70]. However, their focus on social media differs from our study's broader examination of various data sources. Similarly, another review study explored the effectiveness of various AI methods, including facial expressions, images, texts, and emotional chatbots, in detecting emotions and depression, highlighting the versatility of AI in this domain [71]. One more review described machine learning algorithms used for depression detection, with a particular model being the most prevalent [72]. Their findings contribute to the understanding of machine‐learning approaches in depression detection.

In summary, our review study differs from existing ones in terms of focus and findings, offering insights into the fusion of features and their impact on depression detection using deep learning models [61]. Feature fusion, encompassing audio, visual, textual, and other modalities, can enhance the classification accuracy of deep models [60]. However, the selection and combination of features should be tailored to the specific deep model being developed to ensure efficient and accurate depression detection in real‐time settings [73] (Table 1).

**Table 1:** Advantages and limitations of using AI tools for detecting anxiety, depression, and suicidal ideation.

Advantages	Limitations
Rapid detection: When depression and/or anxiety are detected by AI, individuals contemplating suicide can be quickly identified, potentially allowing for prompt intervention.	Ethical and privacy concerns: Research has shown that using AI to detect suicidal ideation may raise ethical challenges, including privacy and confidentiality concerns [64, 74, 75].
Enhanced accuracy: AI tools offer improved accuracy in detecting depression and/or anxiety, aiding in the precise identification of suicidal ideation.	Lack of diversity in data sets: Limited databases reflecting diverse ethnic groups present a disadvantage, potentially limiting the applicability of findings to the global population [76].
Cost‐effectiveness: Utilizing AI tools for diagnosing anxiety and/or depression proves cost‐effective, supporting efficient identification of suicidal ideation.	Uncertainty in identifying suicidal ideation: A review indicates that AI methods for diagnosing anxiety and/or depression may overlook specific characteristics that reliably identify suicidal ideation, which could hinder understanding of this critical aspect [76].

AI techniques have benefits but also drawbacks when it comes to diagnosing depression and/or anxiety. Therefore, the following table analyzes the benefits and drawbacks of this review study.

In this review paper, we explore the potential of AI tools to overcome the drawbacks of conventional diagnostic procedures, based on their usage in diagnosing neurological illnesses, depression, and/or anxiety disorders. EEG signals are evaluated most frequently, followed by audio and/or face video aspects. To find the most useful characteristics for detecting suicidal ideation in patients with depression and/or anxiety disorders, more study is necessary. Future research will make use of these suggested characteristics to provide a more precise evaluation of suicide risk. Additionally, the integration of AI in intraoperative monitoring has been proposed as a method to improve real‐time decision support and reduce surgical complications, illustrating the expanding role of AI applications in diverse clinical settings [77].

## 4 Conclusion

Depression and anxiety disorders rank among the top causes of the global disease burden, specifically first and sixth, respectively. Treating and diagnosing them promptly is still difficult. The use of AI might have a big impact on the psychiatric profession. Streamlining tasks can alleviate the workload for psychiatrists. Chatbots produced by AI are also a fantastic tool for managing mental health conditions. Smartphone apps and social networking make chatbots easily accessible to patients at home. This technology uses taught skills, such as recognizing and correcting cognitive errors, to detect indications of sadness and anxiety. Wearable electronics combined with AI tracking of patient data can lead to early diagnosis and more individualized treatment.

Using a variety of data sources, such as social media and smartphone data, machine learning models, including support vector machines, have been investigated for the diagnosis of anxiety and depression. Our study offers a more thorough investigation of how combining features can enhance depression identification through deep learning models, whereas previous review studies offer insights into alternative techniques. The application of AI to emotional well‐being raises legitimate concerns about ethical conundrums like algorithm bias, privacy concerns, and the uncertainty of identifying suicidal thoughts among patients with depression or anxiety disorders. However, AI has significant advantages like rapid detection, improved precision, and cost‐effectiveness. Innovative therapy modalities, including chatbots like Tess and Woebot, have been developed as a result of recent advances in artificial intelligence.

The subject of mental healthcare could transform thanks to artificial intelligence, notwithstanding its drawbacks. To fully reap the rewards of AI while preserving patient liberty and well‐being, more research and ethical considerations are necessary. Finding the ideal balance between using AI tools to improve treatment outcomes and maintaining the crucial human touch in mental health services is crucial.

## Author Contributions

**Tilyan Kambar and Sara Tariq:** conceptualization, data curation, investigation, methodology, formal analysis, writing – original draft, writing – review and editing. **Saman Shahzad** and **Hajra Sana Sultan:** data curation, formal analysis, investigation, methodology, writing – original draft, writing – review and editing. **Araj Naveed Siddiqui** and **Fakiha Ahmed Shah:** data curation, formal analysis, investigation, methodology, writing – original draft, writing – review and editing. **Hamna Khanani** and **Ayesha Khan:** data curation, formal analysis, investigation, methodology, validation, writing – original draft, writing – review and editing. **Hussain Haider Shah** and **Sameer Abdul Rauf:** data curation, formal analysis, investigation, methodology, writing – original draft, writing – review and editing. **Muhammad Abdul Wasay Zuberi** and **Radeyah Waseem:** data curation, formal analysis, investigation, methodology, visualization, writing – original draft, writing – review and editing. **Muhammad Sheheryar Hussain** and **Md Ariful Haque:** data curation, formal analysis, investigation, visualization, methodology, writing – original draft, writing – review and editing.

## Funding

The authors have nothing to report.

## Ethics Statement

The authors have nothing to report.

## Conflicts of Interest

The authors declare no conflicts of interest.

## Transparency Statement

The corresponding author, Md Ariful Haque, affirms that this manuscript is an honest, accurate, and transparent account of the study being reported; that no important aspects of the study have been omitted; and that any discrepancies from the study as planned (and, if relevant, registered) have been explained.

## Data Availability

Data sharing not applicable to this article, as no data sets were generated or analyzed during the current study.
